# Comparison of Different Phenotypic Tests versus PCR in the Detection of Carbapenemase-Producing *Pseudomonas aeruginosa* Isolates in Hamadan, Iran

**DOI:** 10.1155/2021/5582615

**Published:** 2021-07-16

**Authors:** Masoumeh Beig, Mohammad Taheri, Mohammad Reza Arabestani

**Affiliations:** ^1^Department of Microbiology, Hamadan University of Medical Sciences, Hamadan, Iran; ^2^Nutrition Health Research Center, School of Medicine, Hamadan University of Medical Sciences, Hamadan, Iran

## Abstract

In recent years, the prevalence of carbapenem-resistant *Pseudomonas aeruginosa* isolates has become a worldwide concern. Rapid and accurate detection of carbapenemase-producing *P. aeruginosa* isolates is so important. The aim of this study was to evaluate the performance of the phenotypic methods such as Modified Hodge test (MHT), CarbaNP (CNPt), combined double-disk synergy test (CDDT), and carbapenem inactivation method (CIM) for rapid and accurate detection of clinical carbapenemase production of *P. aeruginosa* isolates. This study was performed on 97 *P. aeruginosa* strains, which were isolated from clinical samples in Hamadan hospitals, western Iran in 2017-2018. Antibiotic susceptibility testing was performed using disk diffusion and minimum inhibitory concentration (MIC) by E-test method. We evaluated the performance of MHT, CarbaNP, CDDT, and CIM tests in comparison to polymerase chain reaction (PCR) for the detection of carbapenemase-producing isolates. Additionally, the presence of carbapenem-resistant genes was investigated using the PCR method. Our findings showed that the highest resistance was to cefoxitin (94.8%). Moreover, among the carbapenem antibiotics, the highest resistance was to imipenem (49.4%). Among the 49 carbapenem-resistant isolates, 42 (85.7%) isolates were MIC positive. The results of phenotypic tests showed that CarbaNP, CIM, CDDT, and MHT tests were positive in (48/49, 97.95%), (46/49, 93.87%), (27/49, 57.44%), and (25/49, 53.19%) of isolates, respectively. CarbaNP and CIM tests showed high sensitivity, specificity, positive predictive values (PPV), and negative predictive values (NPV) compared to PCR in *P. aeruginosa* isolates. CarbaNP and CIM tests are highly sensitive and specific tests for identifying carbapenemase-producing *P. aeruginosa* isolates.

## 1. Introduction

*Pseudomonas aeruginosa* is an opportunistic pathogen and is a major cause of nosocomial infections worldwide [1]. However, carbapenems are considered the last line in the treatment of severe infections caused by *P. aeruginosa* isolates [2]. Over the past decade, the emergence of carbapenem-resistant isolates has become a major concern in health care systems. Although multiple mechanisms of carbapenem resistance have been reported, most of the mechanisms are relevant to the prevalence of carbapenemases enzymes, belonging to Ambler class A (KPCs), class B (VIMs, IMP, SPM, SIM, and GIM), and class D (OXA-48) *β*-lactamases [3]. Bacterial isolates, which are capable of producing carbapenemase enzymes, have the ability to inactivate a wide range of *β*-lactams, including penicillins, cephalosporins, carbapenems, and monobactams [4].

These isolates can spread rapidly in the hospital environment causing nosocomial infections with high mortality. So, rapid identification of carbapenemase-producing *P. aeruginosa* isolates is most important for timely detection, treatment, and performance of infection control measures to prevent the expansion of these resistant isolates [5]. Although molecular methods remain the gold standard for the identification of carbapenemase-producing isolates and enzyme types, carbapenemase genes can be easily detected by PCR [6]. However, for reasons such as the high cost and the inability of this method to detect new carbapenemase genes, various phenotypic methods such as CarbaNP, CIM, MHT, and CDDT have been developed for the rapid detection of carbapenemase-producing isolates [6]. In our study, we investigated the diagnostic values of four phenotypic tests (CarbaNP, CIM, MHT, and CDDT) for the detection of carbapenemase-producing *P. aeruginosa* isolates. CarbaNP and CIM tests are two of the phenotypic tests, which can be done with routine laboratory equipment (in-house) and have been recently recommended by Clinical and Laboratory Standards Institute (CLSI) guidelines for the detection of carbapenemase-producing *P. aeruginosa* isolates [7].

## 2. Materials and Methods

### 2.1. Isolation and Identification of *P. aeruginosa*

This descriptive cross-sectional study was carried out through November 2017 to May 2018. A total of 97 *P. aeruginosa* isolates were collected from hospitalized patients via different clinical specimens including urine, wound, blood, trachea, and other clinical specimens. They were hospitalized in the educational hospitals of Hamadan University of Medical Sciences, Hamadan, Iran. Initially, after the collected samples were transferred to the microbiological laboratory, they were cultured on a blood agar medium and the pure colonies were isolated. Then, Gram staining and various biochemical tests, including oxidase, catalase, oxidative-fermentative test, growth on media including triple sugar iron agar (TSI), Cetrimide agar, and growth at 42°C, were performed to identify *P. aeruginosa* isolates. Finally, the isolates were kept in brain heart infusion (BHI) media containing 20% glycerol and stored at −70°C [8]. This study was approved by the ethics committee of the University of Medical Sciences, Hamadan, Iran (Code No.: IR.UMSHA.REC.1396.662).

### 2.2. Antimicrobial Susceptibility Testing

Antimicrobial susceptibility testing for *P. aeruginosa* isolates was performed according to the CLSI (2017) instructions using the disk diffusion method (Kirby-Bauer) on Mueller–Hinton agar plates to various antibiotics [9]. All disks, including imipenem (10 *μ*g), meropenem (10 *μ*g), doripenem (10 *μ*g), ceftazidime (30 *µ*g), amikacin (30 *μ*g), tetracycline (75 *µ*g), piperacillin/tazobactam (1000/10 *μ*g), piperacillin (100 *μ*g), ceftriaxone (30 *µ*g), cefotaxime (30 *µ*g), cefoxitin (30 *µ*g), and ciprofloxacin (5 *µ*g), were purchased from the British MAST Group (UK). For samples resistant to carbapenem, the MIC test was performed by the imipenem E-test. *P. aeruginosa* ATTC27853 was used as a standard strain.

### 2.3. Selection of *P. aeruginosa* Isolates for Phenotypic and PCR Tests

Clinical isolates based on carbapenem resistance profile were selected and examined for phenotypic and PCR tests.

### 2.4. Phenotypic Detection of Metallo-*β*-lactamase (MBL) by Combined Double-Disk Synergy Test (CDDT)

CDDT was used for phenotypic identification of MBLs producing *P. aeruginosa* isolates. Imipenem (IMP) (10 *μ*g) and imipenem + ethylenediaminetetraacetic acid (EDTA) discs were used to detect MBL-producing *P. aeruginosa* isolates. After 18–24 hours of incubation at 35°C, if the increase in inhibition zone with the imipenem-EDTA disk was ≥7 mm compared to the IMP disk alone, it was considered as MBL positive [10].

### 2.5. Modified Hodge Test (MHT)

This phenotypic test is recommended by CLSI (2017) to detect carbapenemase-producing bacteria. At first, a 0.5 McFarland dilution of *Escherichia coli* (ATCC 25922) in 5 ml of broth or saline was prepared. A 1 : 10 dilution was streaked as lawn on to a Mueller Hinton agar (MHA) plate. A 10 *µ*g ertapenem disk (Mast, UK) was placed on the center of the plate [11]. *P. aeruginosa* isolates (test isolates) were streaked in four different directions. *P. aeruginosa* isolates were cultured in a direct line from the edge of the disc outwards to the periphery of the plate. The plates were then incubated at 37°C for 16 to 24 hours. The cloverleaf-like structure indicated the production of carbapenemase by the test isolates. *Klebsiella pneumoniae* (ATCC BAA-1705) was used as a positive control [12].

### 2.6. Carbapenem Inactivation Method (CIM)

In this test, for each one of the isolates, a 10 *µ*L loop of culture was suspended in a 2 mL tryptic soy broth (TSB) medium. A meropenem disk was added to each tube using a sterile loop. After the incubation of the tubes for 2 hours at 35°C ± 2°C [13], meropenem disk was removed from the suspension, then placed on Mueller–Hinton agar plate, inoculated with a susceptible *E. coli* indicator strain (ATCC 29522), and subsequently incubated at 35°C for 18–24 hours. When the suspected isolates produce the carbapenemase enzymes, the meropenem disc is inactivated and the susceptible indicator strain can grow in the presence of the inactive disc [14].

### 2.7. CarbaNP Test (CNPt)

First, two microcentrifuge tubes were labeled as (a) and (b); then, 100 *µ*l of the bacterial reagent was added to each tube. For each isolate, 1 *µ*L of bacteria from an overnight blood agar plate in both tubes was inoculated. Then, 100 *µ*l of solutions A and B was added to tubes “a” and “b,” respectively, and they were vortexed. The tubes were then incubated at 35°C for up to 2 hours [12]. After 2 hours, the carbapenemase-producing isolates cause the pH shift and produced yellow color, but the isolates that do not produce carbapenemase enzymes remained the same color as the solution [15].

### 2.8. DNA Extraction

Total DNAs of *P. aeruginosa* isolates were extracted by the boiling method. Briefly, 3–5 colonies of overnight bacterial culture were suspended again in 500 *µ*l of sterile distilled water, boiled for 30 mins, and then centrifuged at 14000*g* for 5 mins to pellet cell debris. Then, the extracted DNA was stored at −20°C [16, 17]. The quantity and quality of the DNA were determined using a nano spectrophotometer (NanoDrop ND-1000, Biocompare, San Francisco, USA) and gel electrophoresis.

### 2.9. Detection of Carbapenemase-Encoding Genes by PCR

Carbapenem-resistant *P. aeruginosa* isolates were tested for KPC, IMP, VIM, SIM, GIM, SPM, OXA-48, and AMPC genes by PCR using specific primers (Table 1) [18–21].

### 2.10. Statistical Analysis

SPSS 19 was incorporated in the analysis of the collected data (Chicago, IL, USA). Student's *t*-test was used to analyze numerical data. The statistical significance was *P* < 0.05. The sensitivity and specificity of phenotypic methods were analyzed against PCR as a gold-standard method by the Chi-square test.

## 3. Results

### 3.1. Antimicrobial Susceptibility Testing of *P. aeruginosa* Isolates

The results of antimicrobial susceptibility test on 97 *P. aeruginosa* isolates showed that the highest resistance was to cefoxitin (94.8%, *n* = 92) and the lowest resistance was to piperacillin/tazobactam (39.2%, *n* = 38). Moreover, among the carbapenem antibiotics, the highest resistance was to imipenem (49.4%, *n* = 48) and the lowest resistance was to meropenem (41.2%, *n* = 40). Furthermore, among the 97 *P. aeruginosa* isolates, 49 isolates were resistant and 48 isolates were susceptible to carbapenems (Table 2). Out of 49 (51.51%) carbapenem-resistant isolates, 42 (85.7%) isolates had positive results for MIC (Table 3).

### 3.2. Demographic Characteristics of Evaluated *P. aeruginosa* Isolates and Antibiogram Resistance

Association of the demographic characteristics of evaluated *P. aeruginosa* isolates and antibiogram resistance has been shown in Table 4.

### 3.3. Results of Isolates Containing Different Classes of Carbapenemase

Out of 49 carbapenems-resistant *P. aeruginosa* isolates which were divided into different Ambler groups including KPC (11/49), B (40/49), AmpC (25/49), and OXA-48 (35/49) and while 48 isolates were susceptible to this antibiotic family, these 49 carbapenem-resistant isolates were tested for different phenotypic and PCR tests.

### 3.4. Phenotypic Tests for the Detection of Class A Beta-Lactamase

The highest sensitivity and specificity of phenotypic tests for the detection of KPC gene were related to CarbaNP and CIM tests (100%), although MHT and CDDT tests had lower sensitivity and specificity for the detection of KPC gene (Tables 5 and 6).

### 3.5. Phenotypic Tests for the Detection of Class B Beta-Lactamase

The highest sensitivity and specificity of phenotypic tests for the detection of IMP, VIM, SIM, SPM, and GIM genes were related to CarbaNP and CIM tests (100%) (Tables 5 and 6).

### 3.6. Phenotypic Tests for the Detection of Class C Beta-Lactamase

The highest sensitivity and specificity of phenotypic tests for the detection of *AmpC* gene were related to CarbaNP tests (100%) (Tables 5 and 6).

### 3.7. Phenotypic Tests for the Detection of Class D Beta-Lactamase

The highest sensitivity and specificity of phenotypic tests for the detection of OXA-48 gene were related to CarbaNP tests (97% and 100%, respectively) (Tables 5 and 6).

### 3.8. Detection of Carbapenemase-Encoding Genes by PCR

The results of the PCR method showed that 11 isolates were producer class A (KPC), 40 isolates class B (IMP, VIM, SIM, SPM, and GIM), 25 isolates class C (AmpC), and 35 isolates class D (OXA-48) carbapenemases.

### 3.9. Phenotypic Tests for the Detection of Combination of Classes A, B, C, and D Beta-Lactamases

The performances of four different phenotypic tests were variable for the 35 *P. aeruginosa* isolates carrying the class D carbapenemase (OXA-48). Generally, the highest positive detection was related to CarbaNP (34/35, 97%), with one missing OXA-48-producing isolate as well as one strain harboring both the OXA-48 and AmpC genes. Out of the 35 P. aeruginosa isolates carrying the class D carbapenemase (OXA-48), 20 isolates (70%) were detected by CDDT test; also out of 25 isolates carrying AmpC cephalosporinase gene, 16 isolates (73%) were detected by CDDT test, and this test could not detect 15 isolates carrying OXA-48 gene and 9 isolates carrying AmpC gene. In this regard, the MHT test had the most unpleasant result; it detected only 18 isolates out of the 35 isolates carrying OXA-48 gene (67%) and 14 isolates out of the 25 isolates carrying AmpC cephalosporinase gene (69%) and missed detecting 11 isolates carrying AmpC gene. Out of 49 carbapenemase-producing isolates, 5 isolates had VIM plus KPC genes simultaneously. These isolates were successfully identified by CarbaNP and CIM tests (5/5, 100%), but these isolates can be detected by the MHT test (4/5, 80%) and CDDT test (3/5, 60%) (Tables 5 and 6).

The performance of the phenotypic methods is listed in Table 3. The CarbaNP and CIM tests leading to comparable sensitivities (97% vs. 94%, *P*=0.02), were higher than that of the MHT and CDDT tests (*P* < 0.003). Owing to the improved detection of the OXA-48 gene carrier isolates, the sensitivity of the CarbaNP test was significantly higher than that of the MHT and CDDT tests (97% vs. 67% and 70%, *P* < 0.001). Similarly, the sensitivity of the CarbaNP and CIM tests, increased to 96% and 89%, which was attributed to the improvement in the detection of the AmpC gene carrier isolates. Specificity of the CarbaNP, CDDT, MHT and CIM tests for the detection of AmpC gene were 100%, 100%, 88%, 96% specificity. Taken together, the CarbaNP and CIM tests possessed the best performance for the efficient detection of Carbapenemase-producing isolates, among the four evaluated methods.

### 3.10. Results of Statistical Analysis

In *P. aeruginosa* isolates, there were significant correlations between the disk diffusion method and phenotypic results for carbapenem antibiotics (*P* ≤ 0.001) and between PCR and phenotypic results (*P* ≤ 0.001).

## 4. Discussion

Increased antibiotic resistance to carbapenems among *P. aeruginosa* isolates has become a public health problem. The accurate and rapid detection of carbapenemase producing of *P. aeruginosa* isolates is necessary for appropriate treatment, prevention of spreading, and control of infections. In the last decade, phenotypic methods were extensively used in clinical laboratories for a first-line detection of the isolates producing carbapenemases [22]. These phenotypic methods have different sensitivity and specificity for the detection of carbapenemase-producing *P. aeruginosa* isolates. In the present study, among 97 *P. aeruginosa* isolates, 49 isolates were identified as carbapenemase producers and 48 isolates were noncarbapenemase producers. Four phenotypic methods including MHT, CDDT, CarbaNP, and CIM tests were performed for the detection of carbapenemase-producing isolates. The MHT test is a simple test that is performed to detect carbapenemase-producing isolates. Some studies have revealed that the MHT test has false-negative and false-positive results [23]. In the current study, the sensitivity and NPV of the MHT test for the detection of MBL producers, with 17 missing detections, were (23/40, 70%) and 34%, respectively. Moreover, the MHT test had (18/35, 67%) sensitivity and 45% NPV for the detection of class D (OXA-48) carbapenemase. The results of Pasteran et al. study showed that the MHT test had low sensitivity (78%) and specificity (57%) for the detection of MBL and KPC-producing *P. aeruginosa* isolates [24]. Carvalhaes in 2010 reported 93% sensitivity for the MHT test and showed the possibility of false-positive results in the MHT test [25]. These false-positive results might be due to the porin loss in the cell wall of bacteria [26]. Furthermore, the lowest NPV value was related to MBL production, so this test is not a suitable method for the identification of MBL-producing isolates. In our study, as Varaiya et al. [27] and Murugan et al. [28] studies, the sensitivity of CarbaNP and CIM tests for the detection of group B carbapenemase was 100%. Among the rapid test which is used to identify MBL-producing *P. aeruginosa* isolates, the selection of simple screening tests like the CDDT test is a critical stage to the monitoring of the emerging resistant determinants. In a study, 147 *P. aeruginosa* isolates had MBL genes, and the CDDT test with high sensitivity as a rapid test for class B ambler was introduced [29]. Since the prevalence of the IMP gene in carbapenem-resistant isolates was high, this test can be used in the early screening of these resistant isolates. Due to the low NPV value and sensitivity of the MHT test, this method is not recommended for the identification of carbapenemase-producing isolates.

CDDT test was used for the detection of KPC, MBL, AmpC, and OXA-48 genes, with 68%, 68%, 73%, and 70% sensitivity, respectively. The lowest sensitivity of the CDDT test was related to the detection of MBL, so it is not recommended for the detection of class B carbapenemase-producing isolates.

The CDDT could be very helpful in daily works in the laboratories to achieve rapid detection among *P. aeruginosa* isolates producing KPC and MBL carbapenemase enzymes. The NPV results of the CDDT test for the detection of MBL producers were as low as the MHT test.

The performance of the CIM and CarbaNP tests for the identification of MBL, KPC, AmpC, and OXA-48 gene producers was also evaluated. The overall sensitivity, specificity, PPV, and NPV of the CIM test for KPC-producing isolates were 100%.

In the present study, the sensitivity of the CIM test for the detection of MBL, OXA-48, and AmpC genes was 97%, 94%, and 89%, respectively. Moreover, the specificity of the CIM test for the detection of MBL, OXA-48, and AmpC genes was 100%. In a study by Elif Aktaş et al., the sensitivity and specificity of the CIM test were 78% and 100%, respectively. Elif Aktaş et al. also showed that there was no significant difference in incubation times [30]. Therefore, the CIM test can be a good alternative test for the identification of carbapenemase-producing isolates [31].

In addition to CIM, the CarbaNP test was recommended for epidemiological or infection control purposes by the CLSI and has wide applications due to the high sensitivity and specificity [7]. However, some disadvantages of this method for the detection of carbapenemase-producing isolates are not just the high cost for the preparation of imipenem powder, but also the low sensitivity of this method for the detection of the OXA-type carbapenemases, especially the OXA-48 positive strains [7]. It has been shown that the protein extraction buffer used in the CarbaNP prevented the color change in the reaction known as the buffer effect, which was the leading cause in the identification of the OXA producers.

The CIM test was first recommended by CLSI in 2017. There are some advantages of this test, including low cost, high sensitivity and specificity, and ease of interpretation of results that make it a suitable phenotypic method for the identification of carbapenemase-producing isolates.

According to the results of this study, among the four phenotypic methods, the CarbaNP and the CIM methods can be used for rapid and appropriate detection of carbapenemase-producing isolates to control the infection and prevent the prevalence of these isolates. It can also be used in routine clinical microbiological laboratories.

## 5. Conclusion

The results of this study showed that the CarbaNP and CIM tests have high sensitivity and specificity for identification of carbapenemase-producing *P. aeruginosa* isolates. Due to their ease of use and the rapidity of these methods, these can be used as very suitable methods for the detection of carbapenemase-producing *P. aeruginosa* isolates in clinical laboratories and medical diagnosis.

## Figures and Tables

**Table 1:** PCR primers for the detection of carbapenemase genes.

Carbapenemase genes	Sequence (5′–3′)	Primer	Expected amplicon size (bp)	Reference
IMP	Imp-F	GGA ATA GAG TGG CTT AAY TCT C	188	[18]
	Imp-R	CCA AAC YAC TAS GTT ATC T		
VIM	Vim-F	GAT GGT GTT TGG TCG CAT A	390	[18]
	Vim-R	CGA ATG CGC AGC ACC AG		
GIM-1	GIM-1F	TCG ACA CAC CTT GGT CTG AA	271	[18]
	GIM-2R	AAC TTC CAA CTT TGC CAT GC		
SPM-1	SPM-1F	AAA ATC TGG GTA CGC AAA CG	477	[18]
	SPM-1R	ACA TTA TCC GCT GGA ACA GG		
SIM-1	Sim-1F	TAC AAG GGA TTC GGC ATC G	570	[18]
	Sim-1R	TAA TGG CCT GTT CCC ATG TG		
KPC 1-5	KPC-1F	CATTCAAGGGCTTTCTTGCTGC	538	[19]
	KPC-1R	ACGACGGCATAGTCATTTGC		
AMPC	AMPC-F	CGGCTCGGTGAGCAAGACCTTC	218	[20]
	AMPC-R	AGTCGCGGATCTGTGCCTGGTC		
OXA-48	OXA-48-F	GCTTGATCGCCCTCGATT	281	[21]
	OXA-48-R	GATTTGCTCCGTGGCCGAAA		

**Table 2:** The results of antibiogram testing for *P. aeruginosa* isolate.

Antibiotic	No. resistant (%)	Intermediate	Sensitive	Total
Piperacillin	42 (43.3)	13 (13.4)	42 (43.3)	97
Piperacillin/tazobactam	38 (39.2)	11 (11.3)	48 (49.4)	97
Ceftazidime	40 (41.2)	6 (6.2)	51 (52.6)	97
Aztreonam	50 (51.5)	19 (19.5)	28 (28.9)	97
Amikacin	40 (41.2)	10 (10.3)	47 (48.5)	97
Ciprofloxacin	53 (54.6)	2 (2.1)	42 (43.3)	97
Meropenem	40 (41.2)	5 (5.2)	52 (53.6)	97
Doripenem	45 (46.4)	2 (2.1)	50 (51.5)	97
Cefoxitin	92 (94.8)	1 (1)	4 (4.1)	97
Tetracycline	53 (54.6)	5 (5.2)	39 (40.2)	97
Ceftriaxone	65 (67)	18 (18.6)	14 (14.43)	97
Imipenem	48 (49.4)	1 (1)	48 (49.4)	97

**Table 3:** Results of MIC and different phenotypic tests for carbapenemase genes.

Gene related to carbapenem resistance	MIC imipenem	CDDT	MHT	CIM	CarbaNP
KPC	>32	6/11	5/11	11/11	11/11
IMP	8 to >32	13/20	15/20	20/20	20/20
VIM	8 to >32	10/19	14/19	19/19	19/19
AMPC	8 to>32	16/25	14/25	22/25	24/25
OXA-48	8 to>32	20/35	18/35	33/35	34/35
SIM	8 to >32	3/8	4/8	8/8	8/8
SPM	16	10/17	11/17	17/17	17/17
GIM	8	3/6	3/6	6/6	6/6
KPC + IMP + AMPC	8 to >32	2/2	2/2	2/2	2/2
KPC + AMPC + OXA-48	16	2/3	1/3	3/3	3/3
IMP + VIM + OXA-48	8	3/6	6/6	6/6	6/6
IMP + AMPC + OXA-48	>32	3/5	4/5	4/5	5/5
IMP + KPC + OXA-48	>32	1/1	1/1	1/1	1/1
VIM + KPC + AMPC	>32	1/3	3/3	3/3	3/3
VIM + KPC + AMPC + OXA-48	8	0/1	1/1	1/1	1/1
IMP + VIM + AMPC	4–8	2/4	4/4	4/4	4/4
KPC + OXA-48+VIM + AMPC	>32	1/1	1/1	1/1	1/1
IMP + AMPC	8	8/11	8/11	11/11	11/11
VIM + KPC	16	3/5	4/5	5/5	5/5
Not detected	<0.25-2	44/48	45/48	48/48	48/48

**Table 4:** Demographic characteristics of evaluated *P. aeruginosa* isolates and antibiogram resistance with three main antibiotics.

Characteristics	No. examined	Imipenem resistance		Meropenem resistance		Doripenem resistance	
		No. (%)	*P*-value	No. (%)	*P*-value	No. (%)	*P*-value
*Age*							
0–10	9	5 (55.6)	0.308	5 (55.6)	0.441	5 (55.6)	0.346
11–20	9	5 (55.6)		5 (55.6)		5 (55.6)	
21–30	7	4 (57.1)		3 (42.8)		3 (42.8)	
31–40	7	3 (42.8)		2 (28.6)		2 (28.6)	
41–50	21	9 (42.8)		9 (42.8)		9 (42.8)	
51–60	14	5 (35.7)		4 (28.6)		3 (21.4)	
61–70	19	13 (68.4)		12 (63.1)		13 (68.4)	
71–80	5	2 (40)		2 (40)		3 (60)	
81–90	5	2 (40)		2 (40)		3 (60)	
91–100	1	1 (100)		1 (100)		1 (100)	
Total no.	**97**	**49 (50.5)**		**45 (46.4)**		**47 (48.5)**	

*Gender*							
Male	70	34 (48.6)	0.232	33 (47.1)	0.488	34 (48.6)	0.345
Female	27	15 (55.6)		12 (44.4)		13 (48.1)	
Total no.	**97**	**49 (50.5)**		**45 (46.4)**		**47 (48.5)**	

*Hospital ward*							
Burn	28	19 (67.8)	0.223	19 (67.8)	0.170	18 (64.3)	0.154
Surgery	5	2 (40)		1 (20)		0 (0)	
Pulmonary	11	5 (45.4)		4 (36.4)		3 (27.3)	
Neurology	3	0 (0)		1 (33.3)		1 (33.3)	
Orthopedics	1	0 (0)		0 (0)		1 (100)	
Trauma	13	7 (53.8)		5 (38.4)		6 (46.1)	
NICU	8	2 (25)		1 (12.5)		1 (12.5)	
ICU	14	9 (64.3)		8 (57.1)		10 (71.4)	
Hematology	2	1 (50)		0 (0)		1 (50)	
Infected	12	4 (33.3)		6 (50)		6 (50)	
Total no.	**97**	**49 (50.5)**		**45 (46.4)**		**47 (48.5)**	

*Sample source*							
BC	22	14 (63.6)		12 (54.5)		12 (54.5)	
UC	12	3 (25)	0.082	4 (33.3)	0.401	4 (33.3)	0.243
TC	33	16 (48.5)		14 (42.4)		15 (45.4)	
TA	5	3 (60)		3 (60)		4 (80)	
Wound	15	9 (60)		7 (46.7)		8 (53.3)	
Sputum	6	2 (33.3)		3 (50)		2 (33.3)	
CSF	2	0 (0)		0 (0)		0 (0)	
Fluid	1	1 (100)		1 (100)		1 (100)	
TT	1	1 (100)		1 (100)		1 (100)	
Total no.	**97**	**49 (50.5)**		**45 (46.4)**		**47 (48.5)**	

*Hospitalization period*							
0-10 days	56	20 (35.7)		18 (32.1)		14 (25)	
11-29 days	25	16 (64)		13 (52)		18 (72)	
1-2 months	16	13 (81.2)	0.433	14 (87.5)	0.455	15 (93.7)	0.601
Total no.	**97**	**49 (50.5)**		**45 (46.4)**		**47 (48.5)**	

**Table 5:** Results of sensitivity, specificity, NPV, PPV in CarbaNP versus CIM test.

	No.	CarbaNP				No.	CIM				MIC
		Sensitivity (%)	Specificity (%)	PPV^*∗*^ (%)	NPV^*∗*^ (%)		Sensitivity (%)	Specificity (%)	PPV (%)	NPV (%)	
A	11/11	100	100	100	100	11/11	100	100	100	100	>32
B	40/40	100	100	100	100	39/40	97	100	100	88	8 to.32
C	24/25	96	100	100	96	22/25	89	100	100	88	8 to.32
D	34/35	97	100	100	93	33/35	94	100	100	87	8 to.32
IMP	20/20	100	100	100	100	20/20	100	100	100	100	8 to.32
VIM	19/19	100	100	100	100	19/19	100	100	100	100	8 to.32
SIM	8/8	100	100	100	100	8/8	100	100	100	100	8 to.32
SPM	17/17	100	100	100	100	17/17	100	100	100	100	8 to.32
GIM	21/21	100	100	100	100	21/21	100	100	100	100	8 to.32

^*∗*^NPV: negative predictive values. ^*∗*^PPV: positive predictive values.

**Table 6:** Results of sensitivity, specificity, NPV, and PPV in MHT versus IMP.EDTA test.

	No.	MHT				No.	IMP.EDTA			
		Sensitivity (%)	Specificity (%)	PPV^*∗*^ (%)	NPV^*∗*^ (%)		Sensitivity (%)	Specificity (%)	PPV (%)	NPV (%)
A	5/11	64	95	84	86	6/11	68	88	68	88
B	23/40	70	97	97	34	22/40	68	90	97	33
C	14/25	69	96	96	68	16/25	73	88	89	72
D	18/35	67	93	97	45	20/35	70	87	94	48
IMP	15/20	80	93	90	85	13/20	74	87	83	80
VIM	14/19	79	93	90	85	10/19	67	88	82	76
SIM	4/8	66	95	80	91	3/8	61	87	57	89
SPM	11/17	73	93	89	84	10/17	70	88	80	82
GIM	11/21	67	93	91	73	12/21	70	87	84	75

^*∗*^NPV: negative predictive values. ^*∗*^PPV: positive predictive value.

## Data Availability

The data and material used are available upon request.

## Abbreviations

MHT: Modified Hodge test
CDDT: Combined double-disk synergy test
CIM: Carbapenem inactivation method
MIC: Minimal inhibitory concentration
PCR: Polymerase chain reaction
SIM: Sulfide indole motility
TSI: Triple sugar iron agar
BHI: Brain heart infusion media
EDTA: Ethylenediaminetetraacetic acid
MHA: Muller-Hinton Agar.
